# The Novel Imipramine–Magnesium Complex Exerts Antidepressant-like Activity in Mice Subjected to the Forced Swim Test and the Tail Suspension Test

**DOI:** 10.3390/molecules30030519

**Published:** 2025-01-23

**Authors:** Anna Serefko, Joanna Lachowicz-Radulska, Aleksandra Szopa, Mariola Herbet, Agnieszka Czylkowska, Katarzyna Ignatiuk, Anna Dołoto, Bernadeta Szewczyk, Sylwia Wośko, Andrzej Wróbel, Jarosław Szponar, Piotr Wlaź, Piotr Skałecki, Jan Wróbel, Weronika Słotwińska, Ewa Poleszak

**Affiliations:** 1 Department of Clinical Pharmacy and Pharmaceutical Care, Medical University of Lublin, 1 Chodźki Street, PL 20-093 Lublin, Poland; anna.serefko@umlub.pl (A.S.); aleksandra.szopa@umlub.pl (A.S.); 2 Chair and Department of Toxicology, Medical University of Lublin, 8 Chodźki Street, PL 20-093 Lublin, Poland; mariola.herbet@umlub.pl; 3 Institute of General and Ecological Chemistry, Faculty of Chemistry, Lodz University of Technology, 116 Żeromskiego, PL 90-924 Lodz, Poland; agnieszka.czylkowska@p.lodz.pl; 4 Student Scientific Club, Department of Clinical Pharmacy and Pharmaceutical Care, Medical University of Lublin, 1 Chodźki Street, PL 20-093 Lublin, Poland; katarzynaignatiuk09@gmail.com; 5 Student Scientific Club, Chair and Department of Toxicology, Medical University of Lublin, 8 Chodźki Street, PL 20-093 Lublin, Poland; anna.do@op.pl; 6 Department of Neurobiology, Maj Institute of Pharmacology Polish Academy of Sciences, 12 Smętna Street, PL 31-343 Krakow, Poland; szewczyk@if-pan.krakow.pl; 7 Laboratory of Preclinical Testing, Chair and Department of Applied and Social Pharmacy, Medical University of Lublin, 1 Chodźki Street, PL 20-093 Lublin, Poland; sylwia.wosko@umlub.pl (S.W.); ewa.poleszak@umlub.pl (E.P.); 8 Second Department of Gynecology, Medical University of Lublin, 8 Jaczewskiego Street, PL 20-090 Lublin, Poland; wrobelandrzej@yahoo.com; 9 Clinical Department of Toxicology and Cardiology, Stefan Wyszynski Regional Specialist Hospital in Lublin, 100 Al. Kraśnicka, PL 20-550 Lublin, Poland; jaroslawszponar@umlub.pl; 10 Toxicology Clinic, Medical University of Lublin, 100 Al. Kraśnicka, PL 20-550 Lublin, Poland; 11 Department of Animal Physiology and Pharmacology, Institute of Biological Sciences, Maria Curie-Skłodowska University, 19 Akademicka, PL 20-033 Lublin, Poland; piotr.wlaz@umcs.lublin.pl; 12 Department of Commodity Science and Processing of Raw Animal Materials, University of Life Sciences, 13 Akademicka Street, PL 20-950 Lublin, Poland; piotr.skalecki@up.lublin.pl; 13 Medical Faculty, Medical University of Lublin, 1 Al. Racławickie, PL 20-059 Lublin, Poland; wrobeljan@onet.eu (J.W.); 59175@student.umlub.pl (W.S.)

**Keywords:** imipramine–magnesium complex, antidepressant-like activity, mice, force swim test, tail suspension test, oxidative stress, glutathione peroxidase, glutathione reductase, total antioxidant status

## Abstract

The objective of this study was to assess the antidepressant efficacy of a novel imipramine–magnesium (IMI–Mg) complex in comparison to the administration of imipramine and magnesium individually. The study utilized adult male albino Swiss mice. Behavioral assessments were conducted using the forced swim test (FST) and the tail suspension test (TST). A locomotor activity test was conducted to exclude false positive results in the FST and TST. Moreover, the study assessed oxidative stress levels in the mice subjected to acute environmental stress by measuring glutathione peroxidase, glutathione reductase, total oxidant status, and total antioxidant status. The administration of the IMI–Mg complex at doses of 5, 10, and 20 mg/kg resulted in a reduction in immobility time in both behavioral tests, thereby indicating the antidepressant-like potential of the tested complex, which was similar to the one observed after the administration of these two compounds as separate drug forms. The efficacy of the novel IMI–Mg complex represents a significant advancement and provides a foundation for future research. This innovative agent has the potential to enhance the safety profile of the therapy, streamline the treatment protocol, improve patient satisfaction, and promote adherence to the therapeutic regimen.

## 1. Introduction

Depression belongs to the group of diseases that are frequently difficult to manage with the available monotherapy. Only a partial response to the drugs, quite high disease recurrence rates, a delayed onset of the antidepressant effect, and bothersome side effects are the most common treatment problems that face both patients with depression and their doctors [1,2,3]. The pharmaceutical industry is constantly searching for new solutions to augment the potency of pharmacotherapies while at the same time to improving the benefit–risk ratio of medications. Among novel strategies, drug–drug combinations have garnered considerable interest. The so-called fixed dose combinations (FDC) usually contain active substances that aim at different molecular targets or signaling pathways that are crucial in the pathogenesis of a given disease [4]. Nowadays, polypills have a particular meaning in the management of cardiovascular diseases [5,6,7,8] and HIV/AIDS [9,10], but they are also used in therapies for pain [11,12,13], HCV [14,15], diabetes [16], dyslipidemia [17], malaria [18], tuberculosis [19], and cancer [20]. Apart from polypills, FDC are used, for example, in inhalers to treat asthma [21] and pulmonary obstructive lung disease [22], injections/infusions to treat infections [23] and pain [24], or topical medications for psoriasis [25] and acne [26]. They have also been investigated for therapeutic applications in glaucoma [27] and cystic fibrosis [28]. Recently, dextromethorphan–bupropion [29] and olanzapine–fluoxetine [2] combination pills have been approved for the treatment of major depressive disorders and treatment-resistant depression, respectively. Among the advantages of drugs that contain more than one active substance a number have been mentioned, including a reduced pill burden, a simplified treatment schedule (which is associated with better quality of life), increased patient compliance, a superior efficacy over monotherapy (also through synergy), a diminished risk of side effects (because of lower doses of individual active substances), and reduced costs. The larger the number of pills, the higher risk of non-adherence, medication errors, and instances where patients forget to take their medications. Better adherence/compliance is associated with improved treatment outcomes and a lower risk of treatment failure or relapses of the disease [4,30,31]. Of course, not all active substances can be used in a fixed dose combination due to drug interactions that could be dangerous for the patient [31]. In fact, in the pharmacotherapy of depression, combining selective serotonin reuptake inhibitors (SSRIs) with each other or with serotonin–norepinephrine reuptake inhibitors (SNRIs) or monoamine oxidase inhibitors (MAOIs) may lead to serotonin syndrome. It is better to add an active substance the main mechanism of which is not associated with a significant increase in serotonin levels. Furthermore, concurrent administration of SSRIs and tricyclic antidepressants (TCAs) is not a good choice due to a possible exacerbation of TCA side effects, since SSRIs may inhibit the TCA’s metabolism via cytochrome P450 2D6 [32]. In clinical practice, quetiapine and aripiprazole (atypical antipsychotics) are used as adjunctive treatment for antidepressants [33]. Similarly, esketamine (a nonselective, noncompetitive antagonist of N-methyl-D-aspartate receptors) administered as nasal spray is used in conjunction with oral SSRIs or SNRIs in treatment-resistant depression [34]. Monoamine reuptake inhibitors can also be safely used in combination with antagonists of presynaptic α2-autoreceptors, such as mianserin, mirtazapine, and trazodone [35].

Encouraging results from our previous experiments with the use of the imipramine–zinc complex [36] stimulated us to examine other complexes, including the IMI–Mg one. Magnesium divalent ions, similarly to zinc ions, are able to form stable complexes with other compounds [37,38]. We hypothesized that such a fixed combination may be more effective than co-administration of both agents as separate drug forms. Imipramine, a prototypical TCA, is frequently used in pre-clinical study as a model antidepressant drug; however, its importance in clinical practice has been gradually decreasing due to its side effects (i.e., sedation, hypotension, blurred vision, constipation, dry mouth, and urinary retention), which result from the inhibition of histamine (H1), α_1_-adrenergic, and muscarinic receptors. However, the drug is still available on the pharmaceutical market in many countries [39]. Magnesium compounds are known from their multidirectional activity in mental disorders, including depression [40]. Their supplementation is recommended for patients suffering from different neurological diseases, including migraines, fibromyalgia, anxiety, depression, and epilepsy. Their supplementation is also recommended to reduce the risk of developing mild cognitive impairment [41]. Throughout the years, outcomes of multiple experiments carried out in in vivo animal models of depression (e.g., chronic mild stress, olfactory bulbectomy, immobility stress, and depression resistant to tricyclic antidepressants), as well as results from a range of clinical trials with depressed subjects, have confirmed that the administration of magnesium salts significantly improves depression status (for a review see [42,43]). Furthermore, a magnesium deficiency may lead to the development of depressive/depressive-like symptoms both in humans and rodents [40,44,45]. It has been confirmed many times that lower serum levels of magnesium may be associated with symptoms of depression [46,47], though such a relationship has not been detected in every study [48].

According to available data, modulation of NMDA receptors, serotonergic (5-HT_1A_, 5-HT_2A/2C_) receptors, dopaminergic (D_1_, D_2_) receptors, noradrenergic (α_1_, α_2_) receptors, and γ-aminobutyric acid (GABA) receptors, as well as its influence of the hypothalamic–pituitary–adrenal (HPA) axis, contribute to the antidepressant activity of magnesium supplementation [49]. Thus, the mechanism of magnesium action (similarly to the one attributed to zinc ions) is multifaceted, and is not primarily associated with an increase in serotonin levels. As a consequence, magnesium salts seem to be promising compounds for use in conjugation with conventional antidepressants.

In light of these recent developments, in the present study we wanted to assess the potential antidepressant-like activity of a newly synthesized imipramine–magnesium (IMI–Mg) complex in two recognized behavioral tests in mice, i.e., the forced swim test (FST) and the tail suspension test (TST). Additionally, we wanted to evaluate oxidative stress parameters in the brain of mice treated with the IMI–Mg complex. The use of the IMI–Mg complex compound is an innovation in the field of researching new therapeutic approaches for treating depression. This study determined the effect of IMI–Mg on glutathione peroxidase (GPX), glutathione reductase (GR), total oxidant status (TOS), and total antioxidant status (TAS) in mice subjected to acute environmental stress. The stress inducer in mice was the FST. The FST can generate oxygen free radicals and exacerbate oxidative stress.

## 2. Results

### 2.1. The Antidepressant Potential of the Imipramine–Magnesium Complex in the FST and in the TST in Mice

**Figure 1:**
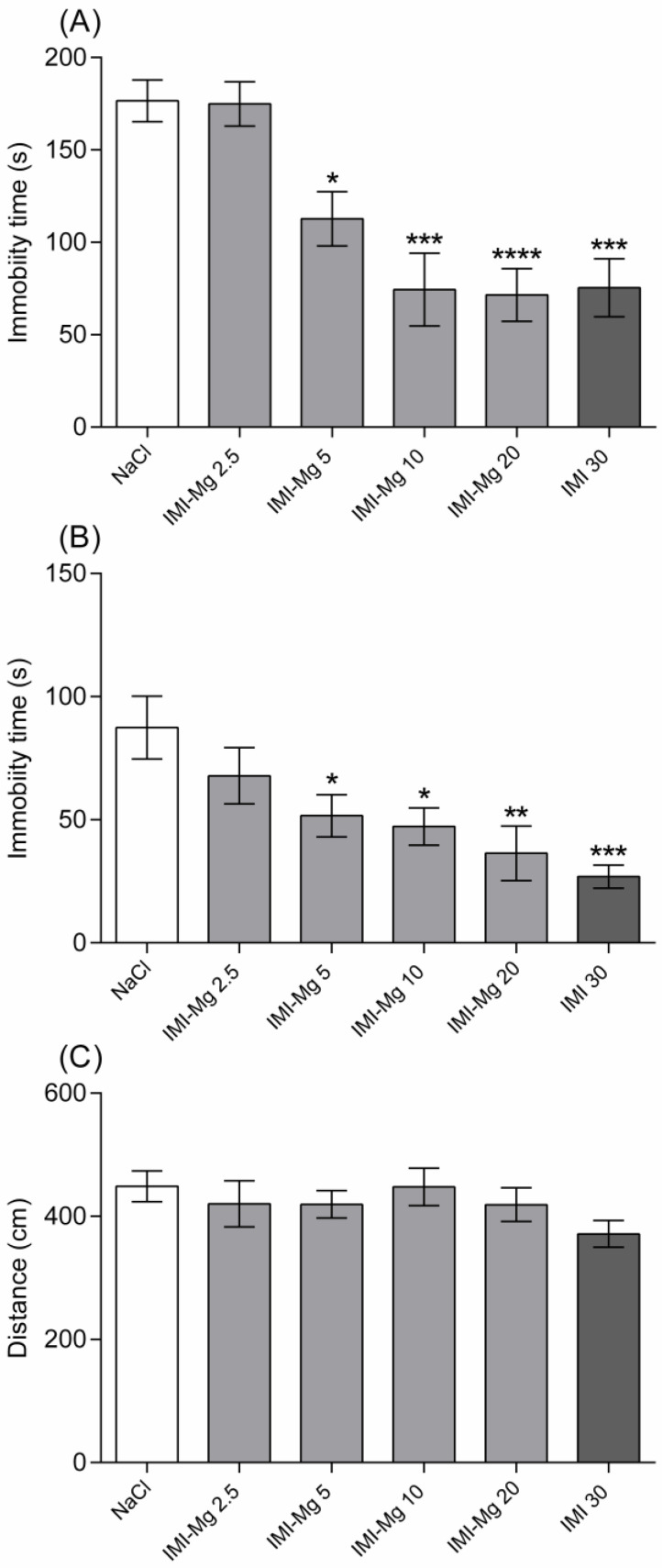
Effects of the novel imipramine–magnesium complex (IMI–Mg) in the forced swim test (**A**), tail suspension test (**B**), and in the actimeter (**C**) in Albino Swiss mice. Saline (a negative control), IMI–Mg (2.5, 5, 10, 20 mg/kg), or imipramine (IMI; 30 mg/kg, a positive control) were administered intraperitoneally 1 h before the behavioral tests. The values represent the mean ± S.E.M. (n = 7–8 animals per group). * *p* < 0.05, ** *p* < 0.01, *** *p* < 0.001, **** *p* < 0.0001 versus saline-treated group (one-way ANOVA, Dunnett’s post-hoc test).

Both in the FST and in the TST the IMI–Mg complex given at doses of 5, 10, and 20 mg/kg significantly reduced the immobility time of tested animals when compared to the saline-treated group. Mice that had received the complex spent a longer time trying to escape the aversive situation (the threat of drowning and being suspended by the tail). The lowest tested dose of the IMI–Mg complex (i.e., 2.5 mg/kg) was ineffective. None of the administered doses of the complex influenced the distance travelled by animals in the actimeter when compared to the control group (Figure 1). One-way ANOVA confirmed significant between-group differences in relation to the results obtained in the FST (F (5, 39) = 11.42; *p* < 0.0001) and in the TST (F (5, 40) = 5.125; *p* = 0.0010), but it did not detect any differences in relation to results obtained in the measurement of the spontaneous locomotor activity (F (5, 42) = 1.019; *p* = 0.4188).

### 2.2. The Antidepressant-like Activity of the Imipramine–Magnesium Complex Versus the Effect of Imipramine and Magnesium Given Either Per Se or Concurrently in the FST and in the TST in Mice

**Figure 2:**
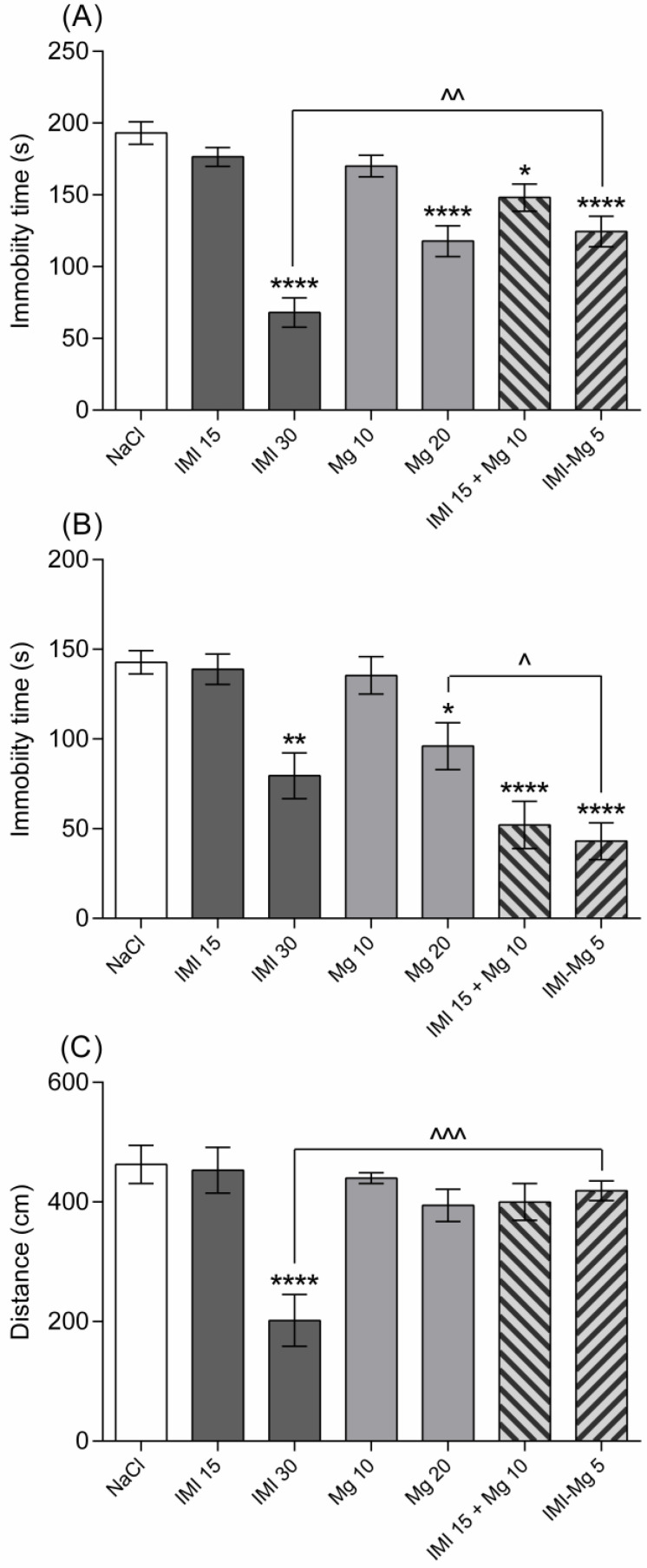
A comparison of the effects of the novel imipramine–magnesium complex (IMI–Mg) versus the effects of imipramine (IMI) and magnesium (Mg) given either per se or concurrently (IMI + Mg) in the forced swim test (**A**), tail suspension test (**B**), and in the actimeter (**C**) in Albino Swiss mice. Saline (a negative control), IMI (15 or 30 mg/kg), Mg (10 or 20 mg/kg), or IMI–Mg (5 mg/kg) were administered intraperitoneally 1 h before the behavioral tests. The values represent the mean ± S.E.M. (n = 8 animals per group). * *p* < 0.05, ** *p* < 0.01, **** *p* < 0.0001 versus saline-treated group, ^ *p* < 0.05, ^^ *p* < 0.01, ^^^ *p* < 0.001 versus IMI–Mg-treated group (one-way ANOVA, Tukey’s post-hoc test).

As presented in Figure 2, the active dose of imipramine in the FST and in the TST was 30 mg/kg, whereas the effective dose of magnesium was 20 mg/kg. Mice that had received the IMI–Mg complex (5 mg/kg) spent a shorter time trying to escape the glass vessel than imipramine-treated animals, but their immobility time was comparable to the one recorded for subjects that were given either magnesium (20 mg/kg) per se or were co-administered with sub-effective doses of imipramine and magnesium (15 mg/kg + 10 mg/kg). In the TST, mice that were injected with the IMI–Mg complex (5 mg/kg) tried to escape the aversive situation for a longer time than animals that were given an effective dose of magnesium (20 mg/kg). The immobility time of mice that received the complex was similar to the one observed for subjects that received either an effective dose of imipramine (30 mg/kg) or co-treatment with sub-effective doses of imipramine and magnesium (15 mg/kg + 10 mg/kg). Imipramine at a dose of 15 mg/kg, magnesium at doses of 10 and 20 mg/kg, co-administration of imipramine and magnesium (15 mg/kg + 10 mg/kg), and the IMI–Mg complex at a dose of 5 mg/kg did not change the spontaneous locomotor activity of the tested rodents when compared to the saline-treated group (*p* > 0.05). Only imipramine given at the active dose of 30 mg/kg significantly reduced the distance travelled by the mice in the actimeter (*p* < 0.0001). One-way ANOVA confirmed significant between-group differences in three behavioural tests: (1) FST: (F (6, 49) = 22.08; *p* < 0.0001), (2) TST (F (6, 46) = 14.44; *p* < 0.0001, and (3) measurement of the spontaneous locomotor activity (F (6, 49) = 8.769; *p* < 0.0001).

### 2.3. Effects of the Imipramine–Magnesium Complex on the Oxidative Stress Parameters

**Figure 3:**
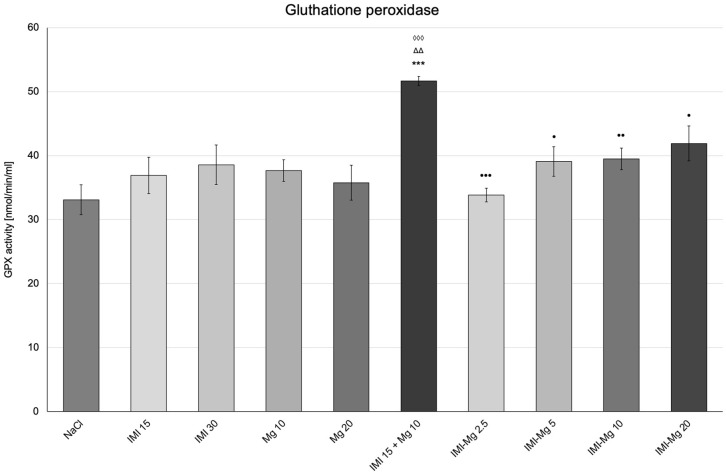
The influence of an intraperitoneal administration of IMI, Mg, IMI + Mg, and IMI–Mg complex on the glutathione peroxidase (GPX) activity. The results are presented as mean ± S.E.M.; *** *p* < 0.001, compared to NaCl control; ^•^ *p* ≤ 0.05, ^••^ *p* < 0.01, ^•••^ *p* < 0.001, compared to IMI + Mg 15 + 10 mg/kg; ^ΔΔ^ *p* < 0.01, compared to Mg 10 mg/kg; ^◊◊◊^ *p* < 0.001 compared to IMI 15 mg/kg (one-way ANOVA, Dunnett’s post-hoc test).

**Figure 4:**
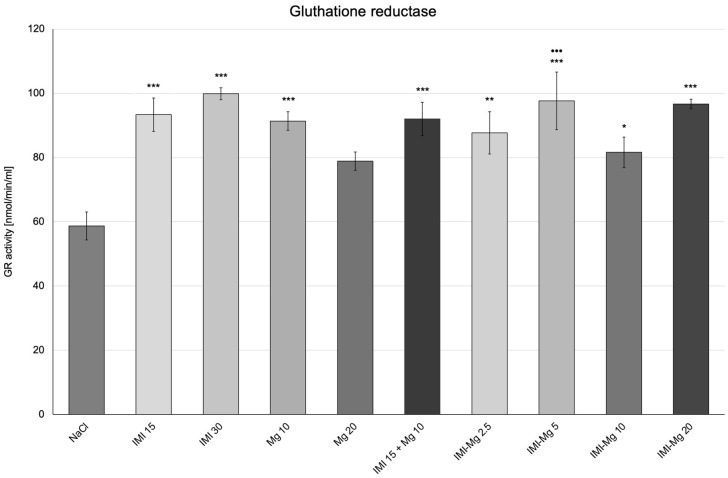
The influence of an intraperitoneal administration of IMI, Mg, IMI + Mg, and IMI–Mg complex on the glutathione reductase (GR) activity. The results are presented as mean ± S.E.M.; * *p* ≤ 0.05, ** *p* < 0.01, *** *p* < 0.001, compared to NaCl control; ^•••^ *p* < 0.001, compared to IMI + Mg 15 + 10 mg/kg (one-way ANOVA, Dunnett’s post-hoc test).

**Figure 5:**
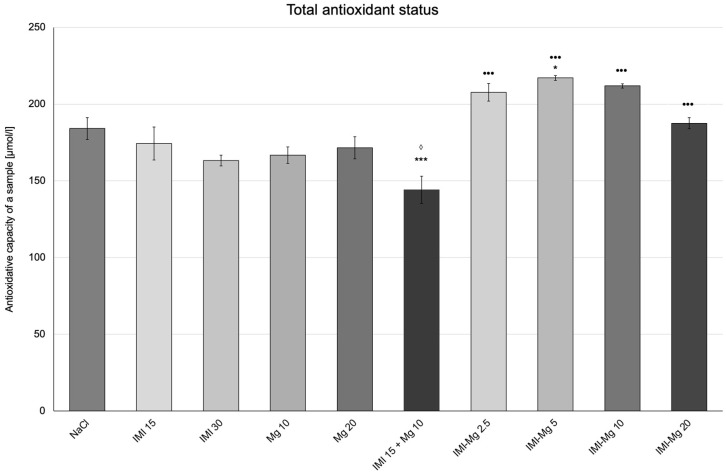
The influence of an intraperitoneal administration of IMI, Mg, IMI + Mg, and IMI–Mg complex on total antioxidant status (TAS). The results are presented as mean ± S.E.M.; * *p* ≤ 0.05, *** *p* < 0.001, compared to NaCl control; ^•••^ *p* < 0.001, compared to IMI + Mg 15 + 10 mg/kg; ^◊^ *p* ≤ 0.05 compared to IMI 15 mg/kg (one-way ANOVA, Dunnett’s post-hoc test).

**Figure 6:**
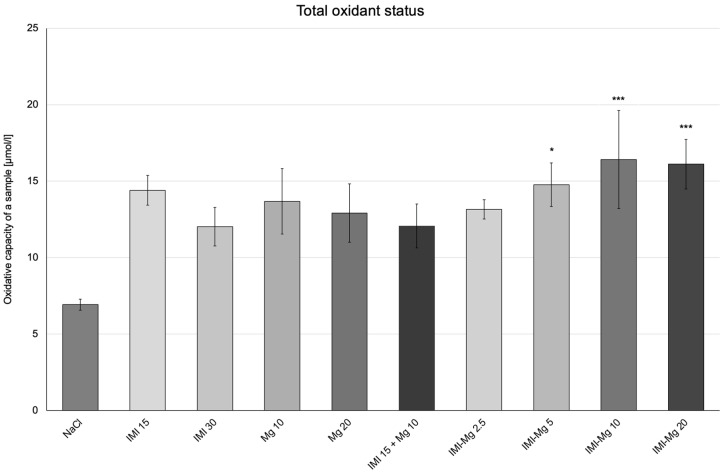
The influence of an intraperitoneal administration of IMI, Mg, IMI + Mg, and IMI–Mg complex on total oxidant status (TOS). The results are presented as mean ± S.E.M.; * *p* ≤ 0.05, *** *p* < 0.001, compared to NaCl control (one-way ANOVA, Dunnett’s post-hoc test).

Effects of the IMI–Mg complex on the oxidative stress parameters are presented in Figure 3, Figure 4, Figure 5 and Figure 6.

#### 2.3.1. Glutathione Peroxidase

In the group of mice that received saline and were exposed to severe environmental stress (FST), the GPX activity was 33.16 ± 2.316 nmol/min/mL (Figure 3). One-way ANOVA confirmed significant between-group differences in GPX activity: F (9, 50) = 2.178; *p* = 0.0396. A statistically significant increase in GPX activity was observed in the group of mice receiving imipramine 15 mg/kg and magnesium 10 mg/kg in combination (*p* < 0.001) as compared to the group that received saline. There were no significant changes in the other groups. Compared with the group of mice receiving imipramine 15 mg/kg and magnesium 10 mg/kg in combination, there was a relevant decrease in GPX activity in groups of mice receiving the IMI–Mg complex at doses of 2.5 mg/kg, 5 mg/kg, 10 mg/kg, and 20 mg/kg (*p* < 0.001, *p* ≤ 0.05, *p* < 0.01, and *p* ≤ 0.05, respectively). A significantly increased GPX activity was also noted in the group that received imipramine 15 mg/kg and magnesium 10 mg/kg in combination as compared to the group receiving magnesium 10 mg/kg and imipramine 15 mg/kg alone (*p* ≤ 0.01 and *p* < 0.001, respectively).

#### 2.3.2. Glutathione Reductase

In the control group, which received a 0.9% NaCl solution, the GR activity was 58.07 ± 4.400 nmol/min/mL (Figure 4). One-way ANOVA confirmed significant between-group differences in GR activity: F (9, 50) = 3.704; *p* = 0.0013. As compared with the group of mice that received saline, there was a statistically significant increase in GR activity in groups of mice receiving: imipramine at 15 mg/kg, imipramine at 30 mg/kg, magnesium at 10 mg/kg, IMI–Mg complex at 2.5 mg/kg, IMI–Mg complex at 5 mg/kg, IMI–Mg complex at 10 mg/kg, IMI–Mg complex at 20 mg/kg, and imipramine 15 mg/kg and magnesium 10 mg/kg in combination (*p* < 0.001, *p* < 0.001, *p* < 0.001, *p* < 0.01, *p* < 0.001, *p* ≤ 0.05, and *p* < 0.001, *p* < 0.001, respectively) as compared to the group that received saline. No significant changes were seen in the group receiving magnesium at 20 mg/kg. A relevant increase was noted in the group that received the IMI–Mg complex at dose of 5 mg/kg (*p* < 0.001), as compared with the group of mice that received imipramine 15 mg/kg and magnesium 10 mg/kg in combination. The other groups showed no significant changes. In the group of animals that received imipramine at 15 mg/kg and magnesium at 10 mg/kg in combination, no changes in GR activity were observed as compared to the groups receiving imipramine at 15 mg/kg or magnesium at 10 mg/kg.

#### 2.3.3. Total Antioxidant Status

In the control group of mice receiving the saline solution, the level of TAS was 184.1 ± 7.155 µmol/L (Figure 5). One-way ANOVA confirmed significant between-group differences in TAS levels F (9, 50) = 5.847; *p* < 0.0001. A statistically significant increase in TAS levels was observed in the groups of mice that received the IMI–Mg complex at 5 mg/kg (*p* ≤ 0.05) as compared to the group that received the 0.9% NaCl solution. There was also a significant decrease in the level of TAS in the group that received imipramine at 15 mg/kg and magnesium at 10 mg/kg in combination (*p* < 0.001) as compared to the control group. No significant changes were observed in the remaining groups. Compared with the group of mice that received imipramine at 15 mg/kg and magnesium at 10 mg/kg in combination, there was a relevant increase of TAS levels in the groups receiving: the IMI–Mg complex at 2.5 mg/kg, the IMI–Mg complex at 5 mg/kg, the IMI–Mg complex at 10 mg/kg and the IMI–Mg complex at 20 mg/kg (*p* < 0.001, *p* < 0.001, *p* < 0.001, and *p* < 0.001, respectively). In the group of mice that received imipramine at 15 mg/kg and magnesium at 10 mg/kg in combination, a slightly significant decrease in TAS level was noted (*p* ≤ 0.05) as compared to the group getting imipramine at 15 mg/kg alone. There were no significant changes compared to the group receiving magnesium at 10 mg/kg.

#### 2.3.4. Total Oxidant Status

In the control group of mice receiving the 0.9% NaCl solution, the level of TOS was 6.929 ± 0.3601 µmol/L (Figure 6). One-way ANOVA confirmed significant between-group differences in TOS levels: F (9, 50) = 2.099; *p* = 0.0472. As compared with the group of mice that received saline solution, there was a significant increase in TOS levels in groups of mice receiving: the IMI–Mg complex at 5 mg/kg, the IMI–Mg complex at 10 mg/kg, and the IMI–Mg complex at 20 mg/kg (*p* ≤ 0.05, *p* < 0.001, and *p* < 0.001, respectively). There were no significant changes in the rest of the groups. In the group of mice receiving IMI at 15 mg/kg and magnesium at 10 mg/kg in combination, no changes in TOS levels were observed compared to the group receiving imipramine at 15 mg/kg and magnesium at 10 mg/kg alone. In the groups of mice that received the IMI–Mg complex at doses of 2.5 mg/kg, 5 mg/kg, 10 mg/kg, and 20 mg/kg, no changes in TOS levels were noted compared to the groups of animals receiving imipramine at 15 mg/kg and magnesium at 10 mg/kg in combination.

## 3. Discussion

As far as we know, this is the first in vivo study that evaluates the antidepressant potential of a newly synthesized IMI–Mg complex. We demonstrated that such a stable combination given at doses of 5, 10, and 20 mg/kg significantly reduced the immobility time of Albino Swiss mice in the FST and in the TST, which indicates that the tested compound has an antidepressant potential. This effect was more pronounced in the FST than in the TST. Furthermore, at the second stage of our experiment, we found out that the lowest active dose of the complex (i.e., 5 mg/kg) was almost similarly effective to 30 mg of imipramine and even a little bit more potent than 20 mg/kg of magnesium ions (in the TST). Outcomes of our study also revealed that the synergistic effect of imipramine and magnesium ions was improved when these two agents were administered in the form of a stable complex. An amount of 5 mg/kg of the tested complex was as effective as 15 mg/kg of imipramine plus 20 mg/kg of magnesium ions co-administered in two separate injections. Importantly, the reactions of mice observed in both behavioral tests were not influenced by spontaneous hyperlocomotion, since the measurement of the locomotor activity of animals did not detect any differences between the distance travelled by the rodents who received the IMI–Mg complex (5, 10, and 20 mg/kg) and the saline-treated group. Additionally, by obtaining the comparable trend in two different behavioral tests (i.e., the FST and the TST) that have non-identical sensitivity to tested compounds, we can be sure that the results were not influenced by environmental factors. Results from the present study are also partially in line with findings from our other projects. We had shown that magnesium ions were able to potentiate the activity of common antidepressant drugs, including citalopram and tianeptine [50]. Additionally, Cardoso and colleagues [51] demonstrated that magnesium chloride improved effects of imipramine, fluoxetine, and bupropion. Magnesium hydroaspartate increased the antidepressant-like effect of DPCPX and istradefylline—selective A_1_ and A_2A_ receptor antagonists, respectively [52]. A similar positive interaction was observed after the co-administration of this magnesium compound and oleamide or AM251, i.e., the CB_1_ cannabinoid receptor ligands [53] as well as with CGP 37849, L-701,324, d-cycloserine, or MK-801, i.e., NMDA antagonists [54]. In studies by Barragan-Rodriguez et al. [55], 450 mg of elemental magnesium/day (given as a 5% solution of magnesium chloride) was as effective as 50 mg/day of imipramine in depressed elderly patients with type 2 diabetes. Other research teams reported that magnesium formulations given as an adjuvant treatment improved clinical outcomes in patients receiving SSRIs [56,57]. The exact molecular mechanism of the IMI–Mg interaction related to their antidepressant effect has not been described yet in detail. Most probably, magnesium ions do not interfere with the passive transport of imipramine [58]. Similarly, it is unlikely that other pharmacokinetic changes are responsible for the synergy detected after co-treatment with imipramine and magnesium. Poleszak et al. [59] did not observe alterations in imipramine, desipramine (an active metabolite of imipramine), or magnesium levels in serum or brains of mice subjected to the joint administration of imipramine and magnesium. Thus, pharmacodynamic processes should be involved in the IMI–Mg interplay.

Apart from evaluating the antidepressant potential of the novel IMI–Mg complex, we also wanted to observe its effects on the oxidative stress parameters. According to the literature data [60], increased oxidative stress plays a crucial role in the development of depression. An elevated generation of reactive oxygen species is associated with alterations of the brain’s structure and its functioning as well as with the activation of pro-inflammatory signaling. The brain is particularly susceptible to ROS production because it metabolizes 20% of the body’s total oxygen and has limited antioxidant capacity. Furthermore, total oxidative status, oxidative stress index, and lipid peroxidation markers are elevated in plasma, serum, or urine in depressed patients [61]. Evidence suggests that high levels of lipid peroxidation in the brain, an important process in the pathogenesis of depression, leads to high levels of oxidative stress [62]. Reduced antioxidant status in depression, characterized by significantly reduced levels of non-enzymatic antioxidant molecules, is usually associated with a decrease in GPX, CAT, and SOD activity in the blood [63]. Therefore, pharmacotherapy for depressive disorders should take into account the role of oxidative stress accompanying the underlying condition and could use substances with antioxidant properties.

In the present study, mice were injected with imipramine or magnesium, either alone or in combination, or with the IMI–Mg complex compound to compare the antioxidative potential of the test agents administered alone and as a complex. The animals used in the study were subjected to the FST after administration with the test substances, which induced physical stress, leading to an increased production of free radicals and resulting in oxidative stress. Compared to the control group, which received a 0.9% NaCl solution, statistically significant changes were obtained in the parameters studied. It was observed that a single administration of the IMI–Mg complex, compared to a combined administration of imipramine and magnesium, statistically significantly reduced GPX activity, caused relevant changes in GR activity, and significantly increased TAS levels. TOS levels were significantly higher in the groups that received the IMI–Mg complex compared to the NaCl control. There are some reports of ROS generation in magnesium deficiency. Zheltova et al. [64] reviewed the evidence that animal tissues with magnesium deficiency show increased vulnerability to lipid peroxidation, which is alleviated by association with antioxidants, implying that free radicals are involved in this process. In animal studies, experimentally caused magnesium deficiency have resulted in depression-like symptoms that were effectively controlled by antidepressants. Magnesium deficiency contributes to oxidative stress by increasing the production of ROS and NO. The main function of GPX is to protect cells against stress, particularly against hydrogen peroxide. This enzyme catalyzes the reaction of hydrogen peroxide and organic peroxides through reduced glutathione. The reaction’s final product is glutathione disulfide (GSSG), which is damaging to cells because it oxidizes the thiol groups of proteins and results in their inactivation. There is a relatively weak antioxidant defense system in the brain. Lipid peroxidation shortens the neuronal lifespan, affects neurotransmitter release, and has been described as a major cause of loss of cellular function under oxidative stress in depression. Lipid peroxidation is one of the major events induced by oxidative stress. It is considered a major consequence of free radical toxicity, leading to profound changes in membrane structure and function that can even cause cell death [65]. In the present study, lipid peroxidation was lower in all groups treated with the IMI–Mg complex compared to the group that received imipramine and magnesium concurrently. This may suggest a reduction in ROS, which contributes to the reduction of oxidative stress damage. Similar results have been obtained by other researchers, who observed an elevation of MDA + 4 HAE in animals with induced depression and a reduction in lipid peroxidation after the administration of antidepressants [66,67]. This reduction in GPX activity may confirm the positive effect of the IMI–Mg complex in inhibiting ROS formation. The decrease in GPX activity indicated that endogenous antioxidant levels were reduced. This may indicate that the production of free radicals was reduced. GPX remains tightly bound with GR, which reconstitutes the reduced form of glutathione. GR plays a crucial role in the cell’s defense against reactive oxygen metabolites, maintaining the cellular pool of reducing GSH efficiently by catalyzing the reduction of GSSG to GSH with accompanying NADPH oxidation. When the complex was administered, there were both significant decreases and increases in GR activity. The increased value was probably due to an increase in oxygen-free radical production. It may suggest a protective effect of the complex directed at maintaining sufficient levels of the reduced form of glutathione and the prevention of hydrogen peroxide accumulation. A decrease in GR activity may result in an insufficient regeneration of GSH. TAS is the serum’s ability to quench free radical production and is defined as an index of the summed activity of all antioxidants. An increase in TAS levels, after the IMI–Mg treatment, suggests a reduction in oxygen-free radicals and an increase in the activity of the antioxidant defense system [68]. In response to ROS overproduction, TAS may be elevated as a compensatory response to restore redox homeostasis. An increase in TAS may indicate an elevation of endogenous antioxidants in the body. It should be noted that the antioxidant capacity of the body also relies on the activity and diversity of proteins with antioxidant properties. Measuring oxidative reaction products provides the most direct evaluation of oxidative stress. As it is not practical to measure the different oxidant molecules separately and their oxidative effects are additive, measuring the TOS is an advantageous solution [69]. However, the experiment showed no significance between the administration of the complex and the administration of single substances. However, TOS levels were significantly higher in the groups that received the IMI–Mg complex compared to the control group administered with a NaCl solution. As mentioned in the discussion, magnesium deficiency can cause oxidative stress, and in our study, groups treated with the IMI–Mg complex showed higher TOS compared to those given the NaCl solution. These changes (especially in the context of the protective role of magnesium) in this respect seem surprising. However, in order to interpret TOS in the experimental context, TAS should be assessed simultaneously, which gives a more complete picture of oxidative balance. In our experiment, we observed increased TAS activity at the same time, which may indicate the involvement of the IMI–Mg complex in antioxidant defense. In the context of assessing two parameters simultaneously, it is difficult to interpret the obtained result of the TOS level unequivocally. Taking into account the results of our experiment, as well as the results of studies by other authors, cited in the discussion section of the paper, it seems that the IMI–MG complex plays an important role in the oxidative–antioxidant balance. The results obtained in our study should be better understood and explained in subsequent experiments. Similar results regarding the antioxidant status of imipramine were obtained by Parachuri et al. [70]. They determined SOD, GPX, lipid peroxidation, and catalase activity. They used the Chronic Mild Stress Method on animals. Imipramine was administered at a dose of 10 mg/kg. The study by Dhingra et al. [71] is also consistent with the present study in terms of the antioxidant status of imipramine. Imipramine at a dose of 15 mg/kg significantly inhibited brain MAO-A activity, reduced plasma nitrite, brain malondialdehyde, and catalase levels, and increased the decreased levels of glutathione in unstressed and stressed mice. The drug significantly reversed the stress-induced increase in plasma corticosterone levels. The study showed promising results with the administration of imipramine and magnesium as complex compounds. However, imipramine is a drug whose therapeutic effects appear after prolonged use. Therefore, it seems reasonable to carry out a study in which the substance is administered over a longer period.

Since magnesium is a natural element for the human body [72], magnesium compounds seem to be a perfect choice as adjunct agents in the treatment of depression. In fact, various magnesium preparations are taken willingly by patients all over the world. Usually, they are ingested in order to relieve muscle cramps and to decrease fatigue and stress [73]. Thus, a single pill combination of magnesium and antidepressant may improve adherence in patients that are afraid of drug side effects. Furthermore, such a magnesium–antidepressant drug complex may extend the usefulness of medications that are effective but the prescribing of which has been limited due to side effects. Generally, magnesium supplements are well-tolerated, with diarrhea as the most frequent undesired reaction. Even though the recommended dietary allowances (RDAs) across the Western countries are from 220 mg/day to 420 mg/day, depending on age [74], the intake of magnesium at a dose 1000 mg/kg (as magnesium oxide) seems to be also relatively safe [75]. In clinical studies with depressed participants, the administered dose of magnesium compounds (magnesium oxide, aspartate, and chloride) varied (for example, in [76,77,78,79,80,81], and it was even up to 2000 mg/kg in [81]. According to the literature data [75], in contrast to hypomagnesemia, hypermagnesemia is an uncommon disorder that usually develops in people with renal dysfunctions (particularly older adults, treated with pump inhibitors or consuming excessive amounts of alcohol), patients with decreased gut motility, and those receiving anticholinergics or opioids. The normal serum magnesium levels are between 1.7 and 2.4 mg/dL, but mild hypermagnesemia (<7 mg/dL) can be either asymptomatic or manifested by non-serious effects (i.e., weakness, nausea, dizziness, and confusion). Only serum magnesium values over 12 mg/dL can be really dangerous for a patient’s life. In our study, the active dose of magnesium ions (given per se) that produced the antidepressant-like effect was 20 mg/kg in mice, which recalculated for humans [82] is ca. 1.62 mg/kg (i.e., 115 mg/kg for a 70-kg adult). The dose of magnesium in the IMI–Mg complex is even lower (it contains ca. 3.28% of magnesium). Of course, this is merely a rough calculation since substances administered in our study were injected intraperitoneally and patients would take the drug orally. Furthermore, it is not possible to directly extrapolate doses from pre-clinical studies into humans, but it is necessary to carry out clinical trials to establish an effective and safe dose range for a given active substance. It should be mentioned that the bioavailability of different magnesium compounds after oral administration is not equivalent. According to Telessy et al. [83], for example, 44.5% of a magnesium aspartate dose is absorbed in the gastrointestinal tract, whereas only 5% of magnesium sulfate is absorbed. Organic salts of magnesium definitely have a better absorption rate than the inorganic forms [84].

Of course, our project has some limitations. First of all, we used only male animals. It is in accordance with common practice when designing experiments related to the assessment of antidepressant-like or anxiolytic-like effects. The estrous cycle could potentially have an impact on mice behavior in the applied tests. However, depression occurs more often in women than in men [85]. As a consequence, female subjects should be included in further studies. Our experiments were acute ones with naïve animals that were not subjected to any depressogenic procedure. In the second (comparison) stage of the study, only one dose of the complex was used. The obtained results should be confirmed in a rodent model of depression in which animals were given the IMI–Mg complex over a longer period (e.g., 2–4 weeks) and, most preferably, at different active doses. Additionally, in our experiments, the IMI–Mg complex was given via the intraperitoneal route as the route of administration that is very frequently used in the preliminary pre-clinical studies. Even though both imipramine and magnesium compounds are absorbed from the alimentary tract after oral administration, further studies should check the bioavailability of the tested complex after oral use.

## 4. Materials and Methods

### 4.1. The Animals

In our study, 208 adult Albino Swiss male mice, which came from a licensed breeder (i.e., the Experimental Medicine Center at the Medical University of Lublin), were used. Animals were randomly assigned to experimental groups of eight subjects. Until the behavioral tests, the mice lived in home cages, with unlimited access to food and water. The cages were placed in a room with a relative humidity of 45–65%, a temperature of 21–24 °C, and artificial light in a 12/12-h day/night cycle. The noise level in the laboratory rooms did not exceed 60 dB, and during the experiments the limit was 35 dB. In order to improve the welfare of the animals, environmental enrichment was used in the cages (houses, cardboard tunnels, wooden blocks, and chew toys for mice). The 3Rs rule as well as binding European law related to experimental studies in animal models were respected. All procedures were approval by the Local Ethical Committee in Lublin (128/2022).

### 4.2. The Tested Substances

The following compounds were used in the study: (1) imipramine hydrochloride (15 and 30 mg/kg, Sigma–Aldrich, Poznań, Poland); (2) magnesium hydroaspartate (10 and 20 mg/kg, calculated as pure magnesium ions; Frampol, Poznań, Poland); and (3) a imipramine–magnesium (IMI-Mg) complex (2.5, 5, 10, and 20 mg/kg, Institute of General and Ecological Chemistry, Medical University of Łódź, Łódź, Poland). The effective and sub-effective doses of imipramine and magnesium ions were selected based on results from previous experiments carried out in our lab [59,86,87]. All tested substances were dissolved in saline (0.9% natrium chloride) immediately before use. The prepared solutions were injected intraperitoneally 1 h before behavioral tests were conducted. The control group received saline. The volume of each injection was 10 mL/kg.

#### The Synthesis of Mg (II) Complex

The [HIMI]^2+^[MgCl_4_]^2−^ compound: 0.8 mmol of magnesium (II) chloride was dissolved in 5 mL of ethanol. An amount of 1.6 mmol of imipramine hydrochloride was dissolved in 20 mL of ethanol and slowly added to the solution of magnesium (II) chloride. The reaction was carried out at room temperature with constant mixing with a magnetic stirrer. After a few days, a white precipitate was formed. After 2 h the precipitate was filtered and washed several times with small amounts of ethanol. Then the product was dried in the open air and analyzed.

The content of the Mg (II) in solid complex was determined by a F-AAS spectrometer with a continuum source of light and an air/acetylene flame (contraAA 300, Analityk Jena AG, Jena, Germany). Absorbance was measured at an analytical spectral line of 285.2 nm. The limit of quantification was 0.04 mg/L. The solid sample was decomposed using the Multiwave 3000 (Anton Paar, Graz, Austria) closed system instrument. The mineralization was carried out for 45 min at 240 °C under a pressure of 60 bar.

### 4.3. The Behavioral Experiments

The behavioral experiments were divided into two stages. At the first stage, the antidepressant potential of the IMI–Mg complex was assessed. At the second stage, the antidepressant-like activity of the IMI–Mg complex was compared with the antidepressant-like activity of imipramine and magnesium given per se as well as with the antidepressant-like activity of imipramine and magnesium when given concurrently. The FST and the TST were carried out. In order to check for false positives in the FST and TST, the spontaneous locomotor activity of all animals was measured.

#### 4.3.1. The Forced Swim Test

The FST was carried out according to the protocol described in the literature [88,89,90]. Animals were placed individually in cylindrical glass vessels (diameter of 10 cm, height of 25 cm) filled with water (water level of 10–12 cm, temperature of 23–25 °C) for 6 min. The immobility of mice was measured after the first 2 min of the test, for 4 min. The first 2 min were treated as a time for the animal to get used to new environmental conditions. Outcomes obtained in the FST were presented as arithmetic means ± S.E.M. for the individual experimental groups.

#### 4.3.2. The Tail Suspension Test

The TST was carried out according to the protocol described in the literature [88,89,90]. Animals were suspended by their tails using an adhesive tape (the adhesive tape was stuck 1 cm from the tip of the tail) for 6 min. The immobility of the mice was measured after the first 2 min of the test, for 4 min. The first 2 min were treated as a time for the animal to get used to new environmental conditions. Outcomes obtained in the TST were presented as arithmetic means ± S.E.M. for the individual experimental groups.

#### 4.3.3. The Spontaneous Locomotor Activity

The spontaneous locomotor activity of animals was measured automatically in OptoVarimex 4 Auto Track (actimeter; Columbus Instruments, Columbus, OH, USA) as we described previously [88,89,90]. Animals were placed individually in the cages of the apparatus for 6 min. Their mobility was measured after the first 2 min of the test, for 4 min, similarly to the time interval applied in the FST and in the TST. The spontaneous locomotor activity of the animals was determined based on the distance (cm) they travelled. Outcomes obtained in this measurement were presented as arithmetic means ± S.E.M. for the individual experimental groups.

### 4.4. Biochemical Analyses

#### 4.4.1. The Preparation of Brain Samples

After performing behavioral tests mice were decapitated. The test material was the prefrontal cortex collected into an Eppendorf tube. The samples were stored at –80 °C until the day of the determination. On the day of the assays, approximately 100–110 mg of the prefrontal cortex was weighed from each brain. The samples were then homogenized for 2 min using Silent Crusher S Homogenizer. After that, the contents of the tubes were centrifuged in a centrifuge at 10,000 G for 15 min. After that time, the supernatant was transferred to test tubes.

#### 4.4.2. Determination of the Biochemical Parameters

Glutathione peroxidase (GPX) activity, glutathione reductase (GR) activity, Total Oxidant Status (TOS), and Total Antioxidant Status (TAS) levels were measured using diagnostic kits. The activity of GPX was measured with the Glutathione Peroxidase Assay Kit (Cayman Chemical, Ann Arbor, MI, USA). GPX is the reaction catalyst for the oxidation of GSH produced by cumene hydroxide. Oxidized GSSG is converted to its reduced form in the presence of GR and NADPH. This is accompanied by the reaction of oxidation of NADPH to NADP+. The reaction intensity is represented as the absorbance at 340 nm. The difference between applied and measured peroxide concentration during a given time is commensurate with the antioxidant reactivity from the sample (antioxidant capacity). The TOS levels were measured using the PerOx^®^ (TOS/TOC) Kit (Immundiagnostik AG, Bensheim, Germany). In this test, total lipid peroxides are measured. Due to the direct correlation between lipid peroxides and oxygen radicals, it is therefore possible to measure and characterize oxidative stress/oxidative status in biological fluids. The difference between measurements 1 and 2 is directly proportional to the peroxide level in the sample.

### 4.5. The Statistical Analysis

The results obtained in our study were statistically analyzed using one-way ANOVA and the Dunett’s or Tukey’s post-hoc test, depending on the experiment. Between-group differences were statistically significant when *p* was below 0.05. The presented data were analyzed using statistical software (GraphPad Prism 10, version 10.0.2).

## 5. Conclusions

To Summarize, outcomes from the present study demonstrated that the combined administration of imipramine and magnesium in a form of a novel IMI–Mg complex has antidepressant potential, which is similar to the that observed after the administration of these too compounds as separate drug forms. Furthermore, the use of the IMI–Mg complex enables a reduction in doses for both substances, which may improve the safety profile of the treatment. In the future, such a procedure may increase patient satisfaction with the prescribed therapy and enhance patient compliance. The obtained results require further evaluation—at first, in different in vivo models of depression, and eventually, in clinical trials. These results cannot be directly extrapolated for the human population, but they may be an inspiration for other scientists.

## Data Availability

The data presented in this study are available in the article.
